# Communication Between US Physicians and Patients Regarding Electronic Cigarette Use

**DOI:** 10.1001/jamanetworkopen.2022.6692

**Published:** 2022-04-15

**Authors:** Cristine D. Delnevo, Michelle Jeong, Arjun Teotia, Michelle M. Bover Manderski, Binu Singh, Mary Hrywna, Olivia A. Wackowski, Michael B. Steinberg

**Affiliations:** 1 Rutgers Center for Tobacco Studies, Rutgers University, New Brunswick, New Jersey; 2 Department of Medicine, Rutgers Robert Wood Johnson Medical School, New Brunswick, New Jersey

## Abstract

**Importance:**

Physicians play a primary role in patient smoking cessation, yet their communication regarding e-cigarettes is not well understood.

**Objective:**

To assess physician-patient communication regarding e-cigarettes.

**Design, Setting, and Participants:**

A national cross-sectional survey in 2018 and 2019 was conducted. Participants were invited by mail; surveys were completed online. Respondents were 2058 board-certified physicians from family medicine, internal medicine, obstetrics and gynecology, cardiology, pulmonary, and oncology. Data were analyzed from August to September 2021.

**Exposures:**

Physician demographic characteristics, tobacco use, medical specialty, and harm-reduction beliefs (ie, not all tobacco products are equally harmful) applied within 2 hypothetical clinical scenarios.

**Main Outcomes and Measures:**

Physicians’ self-reported e-cigarette communication (being asked about e-cigarettes by patients and recommending e-cigarettes to patients) and hypothetical e-cigarette communication in 2 clinical scenarios.

**Results:**

Among 2058 physicians, the mean (SD) age was 51.6 (10.5) years, and 1173 (58.5%) were male. More than 60% of physicians believed all tobacco products to be equally harmful. Overall, 69.8% of physicians reported ever being asked about e-cigarettes by their patients (35.9% in the past 30 days), and 21.7% reported ever recommending e-cigarettes to a patient (9.8% in the past 30 days). Pulmonologists (adjusted odds ratio [aOR], 2.14, 95% CI, 1.10-4.16) and cardiologists (aOR, 2.04; 95% CI, 1.03-4.05), as well as physicians who implemented the US Public Health Service Clinical Practice Guidelines (aOR, 1.77; 95% CI, 1.12-2.80), had greater odds of recommending e-cigarettes to patients. Physicians who endorsed a harm-reduction perspective (aOR, 3.04, 95% CI, 2.15-4.31) and had ever smoked cigarettes (aOR, 1.98; 95% CI, 1.27-3.08) were significantly more likely to recommend e-cigarettes. Physicians who reported being asked about e-cigarettes had greater odds of recommending e-cigarettes (aOR, 16.60; 95% CI, 10.33-26.68). In clinical scenarios, physicians were overall more likely to recommend e-cigarettes for cessation to an older heavy smoker with multiple unsuccessful quit attempts (49.3%; 95% CI, 47.1%-51.4%) than a younger light smoker with no prior cessation treatments (15.2%; 95% CI, 13.6%-16.7%) (*P* < .001).

**Conclusions and Relevance:**

In this survey study of physicians, findings suggest that physicians may recommend switching to e-cigarettes for some patients who smoke cigarettes under certain circumstances, presumably for cessation. The belief that all tobacco products are equally harmful was associated with lower rates of recommending e-cigarettes. As the evidence base grows for e-cigarette efficacy for smoking cessation, there is need for physician education regarding e-cigarette efficacy.

## Key Points

**Question:**

How do physicians communicate with their patients about e-cigarettes?

**Findings:**

In this cross-sectional survey of 2058 respondents, physicians who were asked about e-cigarettes by their patients, endorsed a harm-reduction perspective, or had ever smoked were more likely to recommend e-cigarettes to patients. In hypothetical clinical scenarios, physicians were more likely to recommend e-cigarettes for an older heavy smoker with prior unsuccessful quit attempts and use of pharmacotherapy for a younger light smoker with no prior cessation treatments.

**Meaning:**

Results of this survey study suggest that physicians are recommending e-cigarettes for cessation or harm reduction under certain risk-based circumstances, such as treating patients with tobacco-related disease.

## Teaser

This survey study assesses communications between physicians and patients about e-cigarette use in 2 waves of a large national survey of physicians in the US.

## Introduction

Although progress has been made in reducing smoking over the last several decades, smoking continues to be associated with approximately 480 000 deaths in the US each year,^1^ and effective strategies to reduce this loss are still needed. In 2018, the National Academies of Sciences, Engineering, and Medicine reported varying levels of evidence regarding the risks to youths and the potential benefits of e-cigarettes, such as smoking cessation and harm reduction for adult cigarette smokers.^2^ Although a high number of youths use e-cigarettes,^3^ the data suggest that most adult e-cigarette users are current and former cigarette smokers who turned to e-cigarettes as a less risky alternative to smoking and for smoking cessation despite limited evidence on safety and efficacy.^4,5,6,7,8,9^ A 2020 systematic review on the use of e-cigarettes for smoking cessation found that use of nicotine-containing e-cigarettes was associated with significantly higher quit rates compared with nicotine replacement therapy or behavioral support.^10^ Although the long-term safety of these devices remains unknown, it is likely that they are far less harmful than combusted cigarettes.^11^ For these reasons, e-cigarettes remain a potentially beneficial option for current adult cigarette smokers who are unable or unwilling to quit otherwise.

Physicians play an important role in smoking cessation because they interact with adult smokers on a regular basis and have established physician-patient relationships.^12^ Many physicians report being asked by their patients about e-cigarettes,^13,14,15^ which may be expected given that patients who smoke cite physicians as a trustworthy source of e-cigarette information.^16^ However, how physicians respond to such patient questions is less clear. A 2020 review of the research literature on health care clinicians and e-cigarettes found that professionals, including physicians, held mixed views regarding the safety and role of e-cigarettes in smoking cessation, which was partially explained by different e-cigarette messaging across countries.^17^ In contrast to the US,^18,19^ physicians in the UK are encouraged to consider e-cigarettes as an option for smoking cessation and to support smokers who wish to try them.^20,21,22^

In addition, the few physician studies in the US regarding e-cigarette communications suggest that while physicians were unlikely to recommend e-cigarettes for smoking cessation,^15,23^ they were open to recommending e-cigarettes in the future if data become available suggesting effectiveness.^24^ Although some early data exist regarding e-cigarette physician-patient communications in the US, with 1 exception,^14^ the US studies were limited to local convenience samples^25,26,27^ or single specialties.^28,29,30^ Furthermore, these products, the scientific evidence base, and the policy environment surrounding e-cigarettes are evolving. The aim of this study is to add to the growing body of knowledge about physician-patient communications and recommendations of e-cigarettes using 2 waves of a large national survey of physicians in the US.

## Methods

This study was a national repeated cross-sectional web-push mail survey of physicians in 2018 and 2019. Main outcomes were physicians’ self-reported e-cigarette communication (being asked about e-cigarettes by patients and recommending e-cigarettes to patients) and hypothetical e-cigarette communication in 2 clinical scenarios. Race and ethnicity were determined by participant self-report. Data were analyzed from August to September 2021. The sampling frame was compiled from the American Medical Association Masterfile purchased from Medical Marketing Services and included a random sample of board-certified physicians practicing in various specialties. In each year, a total of 3000 physicians were sampled. In 2018, the sample was distributed evenly across 6 specialties (ie, 500 participants were randomly selected from each specialty): family medicine, internal medicine, obstetrics and gynecology, cardiology, pulmonology, and oncology. In 2019, the sample was distributed evenly across 4 specialties (ie, 750 participants were randomly selected from each specialty): family medicine, internal medicine, obstetrics and gynecology, and pediatrics. Pediatricians were added in 2019 given the rise in e-cigarette use among youths. The Rutgers Biomedical Health Sciences Institutional Review Board approved the study as exempt with a waiver of written consent to facilitate anonymous participation. This study followed the American Association for Public Opinion Research (AAPOR) reporting guideline for survey studies.

Survey fielding occurred from February to July 2018 (wave 1) and April to July 2019 (wave 2). Survey method experiments were embedded in the study in wave 1 to refine the web-push field procedures^31^ and test differing survey incentives ($25 vs $50); wave 2 used a $50 incentive. An initial mailing contained a personalized introductory cover letter, an upfront incentive, and instructions on how to complete the web survey (ie, the survey URL was provided with an anonymous login code). The cover letter explained that the study was to facilitate our understanding of physician beliefs and practices with respect to cigarette smoking cessation, harm reduction, and potential reduced-risk products, such as e-cigarettes. After 1 week from the first mailing, a second mailing contact by postcard was sent to nonrespondents. The third mailing to nonrespondents mirrored the first contact, excluding the gift card. The fourth mailing to all nonrespondents included a paper survey as well as a cover letter with instructions on how to complete the web survey, allowing nonrespondents on the fourth contact to choose the data collection mode (ie, paper survey or web survey).

Using the AAPOR response rate 3 calculation,^32^ which estimates the proportion of cases of unknown eligibility that are eligible, our response rate was 51.8% in 2018 and 59.1% in 2019. Given the focus of this study on physician-patient communications about e-cigarettes in the context of adult cigarette smoking, the analytic sample excluded pediatricians who answered different questions regarding e-cigarettes (ie, prevention focused).

The survey instrument was developed using key domains of interest including but not limited to demographic characteristics, medical background, clinical practice, tobacco treatment practices, harm-reduction beliefs, and e-cigarette patient communication and messaging. Survey items were adapted from previous physician surveys.^14,33^ The results of a previous qualitative study of physicians helped inform question development,^24^ and the instrument underwent cognitive testing. Physicians were presented with 2 hypothetical clinical scenarios and then asked how they would communicate with the specific patient in each scenario about e-cigarettes. The first patient was a young female light smoker who had never tried any cessation methods, and the other patient was an older male heavy smoker with multiple unsuccessful quit attempts using various cessation medications.

### Statistical Analysis

Logistic regression analyses were used to examine factors associated with physicians being asked by patients about e-cigarettes (yes vs no) and physicians recommending electronic cigarettes (yes vs no). Paired *t* tests were used to compare physician responses to 2 hypothetical clinical scenarios. Analyses were performed using Stata/MP, version 17 (StataCorp LLC). Statistical significance was set at 2-tailed *P* < .05.

## Results

The sample contains cross-sectional data from 2058 respondents over 2 waves (2018 and 2019). Table 1 summarizes the demographic and tobacco-related characteristics of the respondents. Overall, 58.5% (95% CI, 56.4%-60.7%) of the respondents were male, with a mean (SD) age of 51.6 (10.5); 12.8% (95% CI, 11.4%-14.4%) of the respondents were Asian or Other Pacific Islander, 4.4% (95% CI, 3.5%-5.3%) were Hispanic, 4.8% (95% CI, 3.9%-5.8%) were non-Hispanic Black, 66.1% (95% CI, 64.0%-68.1%) were non-Hispanic White, and 6.9% (95% CI, 5.8%-8.0%) were South Asian individuals. With respect to medical specialty, both waves included primary care physicians, who constituted most of the sample, with 28.1% (95% CI, 26.2%-30.1%) specializing in family medicine, followed by obstetrics and gynecology (27.0%; 95% CI, 25.1%-28.9%) and internal medicine (22.3%; 95% CI, 20.5%-24.1%), whereas subspecialists, who participated only in wave 1, constituted a smaller percentage as follows: pulmonary (8.6%; 95% CI, 7.4%-9.8%), cardiology (7.2%; 95% CI, 6.1%-8.3%), and oncology (6.9%; 95% CI, 5.8%-8.0%).

**Table 1.** Summary of Demographic Characteristics of 2058 Respondents

Characteristic	No.	% (95% CI)
Age, mean (SD), y	51.6 (10.5)	NA
Sex		
Male	1173	58.5 (56.4-60.7)
Female	831	41.5 (39.3-43.7)
Race and ethnicity^a^		
Asian or Other Pacific Islander	256	12.8 (11.4-14.4)
Black	96	4.8 (3.9-5.8)
Hispanic	88	4.4 (3.5-5.3)
South Asian	138	6.9 (5.8-8.0)
White	1318	66.1 (64.0-68.1)
Other	99	5.0 (4.0-5.9)
Specialty		
Family medicine	574	28.1 (26.2-30.1)
OB/GYN	551	27.0 (25.1-28.9)
Internal medicine	455	22.3 (20.5-24.1)
Pulmonary	175	8.6 (7.4-9.8)
Cardiology	147	7.2 (6.1-8.3)
Oncology	140	6.9 (5.8-8.0)
Year of data collection		
Wave 1/2018	1058	51.4 (49.3-53.6)
Wave 2/2019	1000	48.6 (46.4-50.8)
PHS awareness		
Unaware	650	32.5 (30.5-34.6)
Aware, not read	811	40.6 (38.4-42.8)
Read, not used	146	7.3 (6.2-8.4)
Uses	392	19.6 (17.9-21.4)
Risk continuum		
All forms of tobacco equally harmful	1222	60.1 (58.0-62.3)
Cigarettes are the most dangerous	810	39.9 (37.7-42.0)
Smoked at least 100 cigarettes		
Yes	253	12.5 (11.1-14.0)
No	1765	87.5 (86.0-88.9)
Ever used e-cigarettes		
Yes	71	3.5 (2.7-4.3)
No	1946	96.5 (95.7-97.3)

Abbreviations: NA, not available; OB/GYN, obstetrics and gynecology; PHS, Public Health Service.

^a^ Race and ethnicity were determined by participant self-report. “Other” was a choice on the survey that participants self-selected, and no further breakdown is available.

Among all respondents, 1 of 3 reported being unaware of Public Health Service Clinical Practice (PHS) guidelines on tobacco at the time of the survey, while 19.6% (95% CI, 17.9%-21.4%) reported using the guidelines. Most physicians (60.1%) endorsed the belief that all forms of tobacco were equally harmful and that cessation from all tobacco use was the best approach, whereas 39.9% endorsed the belief that getting smokers to stop smoking cigarettes should be the goal, even if it meant switching to less harmful forms of tobacco. Ever regular cigarette smoking among physicians was 12.5% (95% CI, 11.1%-14.0%), and 3.5% (95% CI, 2.7%-4.3%) indicated they had tried an e-cigarette.

Table 2 details physician-patient communications regarding e-cigarettes. Overall, 69.8% (95% CI, 67.8%-71.8%) of physicians reported ever being asked about e-cigarettes by their patients, and 35.9% (95% CI, 33.8%-38.0%) reported being asked about e-cigarettes in the 30 days preceding the survey. In addition, 21.7% (95% CI, 19.9%-23.5%) of physicians reported ever recommending e-cigarettes to a cigarette smoker, and 9.8% (95% CI, 8.5%-11.0%) reported making such a recommendation in the past 30 days. Overall, male physicians were more likely to report being asked about e-cigarettes in the past 30 days (40.3%; 95% CI, 37.5%-43.1%) than female physicians (30.5%; 95% CI, 27.3%-33.6%); *P* < .001). Male physicians were also more likely to report having recommended e-cigarettes in the past 30 days (12.1%; 95% CI, 10.2%-13.9%) than female physicians (6.6%; 95% CI, 4.9%-8.3%) (*P* < .001). Black physicians were least likely to report ever being asked about e-cigarettes by their patients (49.5%; 95% CI, 39.2%-59.7%; *P* < .001), whereas Hispanic physicians were most likely to report being asked about e-cigarettes within the past 30 days (47.7%; 95% CI, 37.1%-58.4%; *P* < .001). There were no significant differences in recommendations by physician race and ethnicity. Pulmonologists and family medicine physicians were most likely to report having ever been asked about e-cigarettes than other specialists (90.3%; 95% CI, 85.9%-94.7% and 84.6%; 95% CI, 81.6%-87.6%; *P* < .001) as well as in the past 30 days (59.4%; 95% CI, 52.1%-66.8% and 47.6%; 95% CI, 43.4%-51.7%; *P* < .001). Pulmonologists were also most likely to recommend e-cigarettes in the past 30 days (17.2%; 95% CI, 11.6%-22.9%; *P* = .001), followed by cardiologists and family medicine physicians (12.9%; 95% CI, 7.4%-18.4% and 10.6%; 95% CI, 8.1%-13.2%). Notably, although cardiologists were less likely to be asked about e-cigarettes than family medicine physicians, our data showed that cardiologists were more likely to recommend e-cigarettes to patients in the past 30 days.

**Table 2.** Patient Prompting About e-Cigarettes and Physician e-Cigarette Recommendation (2018-2019)

Variable	Asked about e-cigarette by patients				Recommended e-cigarettes to patients			
	Ever		In past 30 d		Ever		In past 30 d	
	% (95% CI)	*P* value	% (95% CI)	*P* value	% (95% CI)	*P* value	% (95% CI)	*P* value
Overall	69.8 (67.8-71.8)		35.9 (33.8-38.0)		21.7 (19.9-23.5)		9.8 (8.5-11.0)	
Sex								
Male	73.0 (70.5-75.6)	.001	40.3 (37.5-43.1)	<.001	25.2 (22.8-27.8)	<.001	12.1 (10.2-13.9)	<.001
Female	65.9 (62.7-69.1)		30.5 (27.3-33.6)		17.2 (14.6-19.8)		6.6 (4.9-8.3)	
Race and ethnicity^a^								
Hispanic	73.9 (64.5-83.2)	<.001	47.7 (37.1-58.4)	<.001	15.9 (8.1-23.7)	.16	6.8 (1.4-12.2)	.49
Asian or Other Pacific Islander	60.9 (54.9-67.0)		23.6 (18.4-28.9)		17.9 (13.2-22.7)		7.8 (4.5-11.2)	
Black	49.5 (39.2-59.7)		24.0 (15.3-32.7)		16.7 (9.1-24.3)		6.3 (1.3-11.2)	
South Asian	72.5 (64.9-80.0)		39.9 (31.6-48.1)		21.2 (14.2-28.1)		11.0 (5.7-16.2)	
White	73.1 (70.7-75.5)		38.6 (36.0-41.3)		23.6 (21.3-25.9)		10.5 (8.8-12.1)	
Other	63.6 (54.0-73.3)		32.3 (22.9-41.7)		21.2 (13.0-29.4)		11.1 (4.8-17.4)	
Specialty								
OB/GYN	50.1 (45.9-54.3)	<.001	20.9 (17.5-24.3)	<.001	15.1 (12.1-18.1)	<.001	7.1 (4.9-9.2)	.001
Cardiology	72.1 (64.8-79.4)		30.3 (22.8-37.9)		24.8 (17.7-31.9)		12.9 (7.4-18.4)	
Family medicine	84.6 (81.6-87.6)		47.6 (43.4-51.7)		24.9 (21.3-28.4)		10.6 (8.1-13.2)	
Internal medicine	72.5 (68.3-76.6)		36.8 (32.3-41.2)		22.7 (18.8-26.5)		9.5 (6.8-12.3)	
Oncology	52.9 (44.5-61.2)		22.3 (15.3-29.3)		14.4 (8.5-20.3)		5.0 (1.3-8.7)	
Pulmonary	90.3 (85.9-94.7)		59.4 (52.1-66.8)		33.1 (26.0-40.2)		17.2 (11.6-22.9)	
PHS awareness								
Unaware	64.6 (60.9-68.3)	<.001	29.8 (26.3-33.4)	<.001	18.3 (15.3-21.3)	.01	7.7 (5.6-9.8)	.02
Aware, not read	67.8 (64.6-71.0)		32.8 (29.6-36.0)		22.5 (19.6-25.3)		9.5 (7.5-11.5)	
Read, not used	71.2 (63.8-78.7)		42.5 (34.4-50.6)		20.1 (13.5-26.8)		10.4 (5.4-15.5)	
Uses	83.4 (79.7-87.1)		52.6 (47.6-57.5)		27.0 (22.6-31.4)		13.6 (10.2-17.0)	
Risk continuum								
All forms of tobacco equally harmful	67.9 (65.2-70.5)	.01	35.1 (32.4-37.8)	.33	13.3 (11.4-15.2)	<.001	6.0 (4.7-7.3)	<.001
Cigarettes are the most dangerous	73.1 (70.0-76.1)		37.2 (33.9-40.6)		34.1 (30.8-37.4)		15.1 (12.6-17.6)	
Smoked at least 100 cigarettes								
Yes	70.4 (64.7-76.0)	.83	36.5 (30.5-42.5)	.89	28.8 (23.1-34.5)	.01	16.7 (12.1-21.4)	<.001
No	69.7 (67.5-71.8)		36.1 (33.8-38.3)		20.8 (18.9-22.7)		8.6 (7.3-9.9)	
Ever used e-cigarettes								
Yes	73.2 (62.7-83.8)	.52	43.7 (31.8-55.5)	.18	40.0 (28.2-51.8)	<.001	18.6 (9.2-27.9)	.01
No	69.6 (67.6-71.7)		35.8 (33.7-38.0)		21.1 (19.3-22.9)		9.3 (8.0-10.6)	
Year of data collection								
Wave 1/2018	69.9 (67.1-72.7)	.93	35.2 (32.3-38.1)	.50	22.2 (19.7-24.7)	.59	10.0 (8.2-11.8)	.74
Wave 2/2019	69.7 (66.9-72.6)		36.6 (33.6-39.6)		21.2 (18.7-23.8)		9.5 (7.7-11.4)	

Abbreviations: OB/GYN, obstetrics and gynecology; PHS, Public Health Service.

^a^ Race and ethnicity were determined by participant self-report. “Other” was a choice on the survey that participants self-selected, and no further breakdown is available.

Physicians unaware of PHS guidelines were less likely to recommend e-cigarettes to patients, with no significant difference in being asked about e-cigarettes in the past 30 days across PHS categories. Although no associations were found between harm-reduction beliefs and being asked about e-cigarettes by patients, the association between physicians’ harm-reduction beliefs and their e-cigarette recommendation practices was significant. Physicians who believed that cigarettes were the most dangerous tobacco product were significantly more likely to report ever recommending e-cigarettes (34.1% vs 13.3%; *P* < .001) as well as in the past 30 days (15.1% vs 6.0%; *P* < .001) compared with physicians who believed all forms of tobacco were equally harmful. Physicians who reported ever smoking, as well as trying e-cigarettes, were more likely to recommend e-cigarettes compared to nonsmokers or those that had not tried e-cigarettes.

Factors associated with being asked about e-cigarettes by patients are reported in Table 3. Increasing physician age (adjusted odds ratio [aOR], 0.98; 95% CI, 0.97-0.99) and Asian or Other Pacific Islander descent (aOR, 0.42; 95% CI, 0.30-0.59) were significantly associated with being less likely to report being asked by their patients about e-cigarettes, and male physicians (aOR, 1.50; 95% CI, 1.20-1.89) were significantly more likely to report being asked about e-cigarettes. Specialty was significantly associated with reporting being asked about e-cigarettes: pulmonologists had the highest odds of being asked (aOR, 5.03; 95% CI, 3.26-7.76), followed by family medicine physicians (aOR, 3.09; 95% CI, 2.34-4.08), internal medicine physicians (aOR, 2.18; 95% CI, 1.61-2.95), and cardiologists (aOR, 1.85; 95% CI, 1.15-3.00). Physician awareness and implementation of the PHS guidelines was a factor associated with physicians reporting they were asked about e-cigarettes (aOR, 2.30; 95% CI, 1.72-3.06). In addition, being asked about e-cigarettes in the 30 days preceding the survey was significantly higher in 2019 than in 2018 (aOR, 1.29; 95% CI, 1.02-1.63).

**Table 3.** Factors Associated With Being Asked by Patients About e-Cigarettes and Recommending e-Cigarettes to Patients in Past 30 Days

Variable	aOR (95% CI)		
	Asked about e-cigarettes	Recommended e-cigarettes to patients	
	Model 1^a^	Model 2^a^	Model 3^a^
Age	0.98 (0.97-0.99)	1.01 (1.00-1.03)	1.03 (1.01-1.05)
Sex			
Male	1.50 (1.20-1.89)	1.58 (1.09-2.29)	1.36 (0.92-2.03)
Female	1 [Reference]	1 [Reference]	1 [Reference]
Race and ethnicity^b^			
Hispanic	1.22 (0.77-1.95)	0.60 (0.26-1.42)	0.45 (0.20-1.03)
Asian or Other Pacific Islander	0.42 (0.30-0.59)	0.80 (0.46-1.39)	1.49 (0.81-2.72)
Black	0.62 (0.37-1.02)	0.97 (0.40-2.32)	1.19 (0.47-3.04)
South Asian	0.86 (0.59-1.26)	1.20 (0.64-2.24)	1.40 (0.73-2.67)
White	1 [Reference]	1 [Reference]	1 [Reference]
Other	0.74 (0.46-1.19)	1.61 (0.81-3.20)	1.74 (0.76-4.00)
Specialty			
OB/GYN	1 [Reference]	1 [Reference]	1 [Reference]
Cardiology	1.85 (1.15-3.00)	2.04 (1.03-4.05)	1.65 (0.76-3.62)
Family medicine	3.09 (2.34-4.08)	1.52 (0.95-2.43)	0.79 (0.48-1.32)
Internal medicine	2.18 (1.61-2.95)	1.21 (0.73-1.99)	0.73 (0.42-1.26)
Oncology	1.15 (0.69-1.92)	0.79 (0.32-1.96)	0.62 (0.24-1.60)
Pulmonary	5.03 (3.26-7.76)	2.14 (1.10-4.16)	0.94 (0.46-1.89)
PHS awareness			
Unaware	1 [Reference]	1 [Reference]	1 [Reference]
Aware, not read	1.06 (0.83-1.34)	1.24 (0.84-1.83)	1.14 (0.74-1.75)
Read, not used	1.83 (1.21-2.76)	1.46 (0.80-2.70)	1.01 (0.51-1.98)
Uses	2.30 (1.72-3.06)	1.77 (1.12-2.80)	1.12 (0.69-1.81)
Endorses risk continuum			
Yes	0.99 (0.80-1.21)	2.58 (1.86-3.57)	3.04 (2.15-4.31)
No	1 [Reference]	1 [Reference]	1 [Reference]
Smoked at least 100 cigarettes			
Yes	0.97 (0.71-1.33)	1.71 (1.14-2.58)	1.98 (1.27-3.08)
No	1 [Reference]	1 [Reference]	1 [Reference]
Ever used e-cigarettes			
Yes	1.36 (0.80-2.30)	1.67 (0.87-3.21)	1.70 (0.83-3.48)
No	1 [Reference]	1 [Reference]	1 [Reference]
Year of data collection			
Wave 1/2018	1 [Reference]	1 [Reference]	1 [Reference]
Wave 2/2019	1.29 (1.02-1.63)	1.15 (0.78-1.70)	1.04 (0.69-1.57)
Patient prompt			
Yes	NI	NI	16.60 (10.33-26.68)
No	NI	NI	1 [Reference]

Abbreviations: aOR, adjusted odds ratio; NI, not included; OB/GYN, obstetrics and gynecology; PHS, Public Health Service.

^a^ Observations: model 1 = 1880; model 2 = 1882; and model 3 = 1887.

^b^ Race and ethnicity were determined by participant self-report. “Other” was a choice on the survey that participants self-selected, and no further breakdown is available.

With respect to recommending e-cigarettes to their patients, we ran 2 models (Table 3), 1 of which controlled for whether or not a patient had asked the physician about e-cigarettes or patient prompting. When patient prompting (aOR, 16.60, 95% CI, 10.33-26.68) was added to the model, male sex, physician specialty, and engagement with the PHS guidelines were not significant factors associated with e-cigarette recommendations. Having ever been a regular smoker (aOR, 1.98; 95% CI, 1.27-3.08) and endorsing a harm-reduction perspective (aOR, 3.04; 95% CI, 2.15-4.31) were significant factors associated with e-cigarette recommendations, even when controlling for patient prompting. Pulmonologists (aOR, 2.14, 95% CI, 1.10-4.16) and cardiologists (aOR, 2.04; 95% CI, 1.03-4.05), as well as physicians who implemented the US Public Health Service Clinical Practice Guidelines (aOR, 1.77; 95% CI, 1.12-2.80) had greater odds of recommending e-cigarettes to patients.

Table 4 presents differences in physician communication in the patient clinical scenario by medical specialty. Overall, physicians were most likely to communicate that they preferred the patient to use US Food and Drug Administration (FDA)–approved pharmacotherapy over an e-cigarette to help them quit. This message was endorsed more often in the scenario of the patient being a young female light smoker (82.1%; 95% CI, 80.4%-83.7%) than an older male heavy smoker with multiple failed quit attempts (56.8%; 95% CI, 54.7%-58.9%; *P* < .001). Similarly, physicians were more likely to encourage smokers to try e-cigarettes to transition away from cigarettes when the patient was a heavy smoker with multiple failed quit attempts (49.3%; 95% CI, 47.1%-51.4%) than if the patient had never tried quitting before (15.2%; 95% CI, 13.6%-16.7%; *P* < .001). Approximately 1 in 4 physicians communicated that e-cigarettes were harmful and discouraged use; this communication was significantly more likely with the younger light smoker than the older heavier smoker (31.7%; 95% CI, 29.7%-33.8% vs 24.9%; 95% CI, 23.1%-26.8%; *P* < .001). This tailored risk-based communication was consistent across medical specialties.

**Table 4.** Messaging to Smokers Regarding e-Cigarettes by Medical Specialty and Patient Characteristics

Variable	Support e-cigarettes to transition away from cigarettes			Prefer patient to use FDA-approved pharmacotherapy rather than e-cigarettes			e-Cigarettes are not effective for cessation and discourage their use			e-Cigarettes are harmful and discourage their use		
	% (95% CI)		*P* value	% (95% CI)		*P* value	% (95% CI)		*P* value	% (95% CI)		*P* value
	29-y Female light smoker^a^	65-y Male heavy smoker^b^		29-y Female light smoker^a^	65-y Male heavy smoker^b^		29-y Female light smoker^a^	65-y Male heavy smoker^b^		29-y Female light smoker^a^	65-y Male heavy smoker^b^	
Overall	15.2 (13.6-16.7)	49.3 (47.1-51.4)	<.001	82.1 (80.4-83.7)	56.8 (54.7-58.9)	<.001	19.0 (17.3-20.7)	17.5 (15.9-19.2)	.06	31.7 (29.7-33.8)	24.9 (23.1-26.8)	<.001
Cardiology	23.1 (16.2-30.0)	53.1 (44.9-61.2)	<.001	71.4 (64.0-78.8)	50.3 (42.2-58.5)	<.001	15.6 (9.7-21.6)	15.0 (9.1-20.8)	.81	25.9 (18.7-33.0)	16.3 (10.3-22.4)	.002
Family medicine	15.7 (12.7-18.7)	49.8 (45.7-53.9)	<.001	85.0 (82.1-87.9)	61.3 (57.3-65.3)	<.001	19.7 (16.4-22.9)	18.8 (15.6-22.0)	.50	34.1 (30.3-38.0)	28.6 (24.9-32.3)	.001
Internal medicine	14.3 (11.1-17.5)	48.8 (44.2-53.4)	<.001	82.4 (78.9-85.9)	56.3 (51.7-60.8)	<.001	20.0 (16.3-23.7)	18.7 (15.1-22.3)	.43	32.3 (28.0-36.6)	23.1 (19.2-27.0)	<.001
OB/GYN	13.8 (10.9-16.7)	47.5 (43.4-51.7)	<.001	83.7 (80.6-86.8)	55.0 (50.8-59.2)	<.001	17.6 (14.4-20.8)	15.8 (12.7-18.8)	.24	30.9 (27.0-34.7)	25.8 (22.1-29.4)	.001
Oncology	12.9 (7.2-18.5)	46.4 (38.1-54.8)	<.001	83.6 (77.4-89.8)	60.0 (51.8-68.2)	<.001	16.4 (10.2-22.6)	17.9 (11.4-24.3)	.59	24.3 (17.1-31.5)	20.7 (13.9-27.5)	.17
Pulmonary	14.9 (9.5-20.2)	56.6 (49.2-64.0)	<.001	79.4 (73.4-85.5)	55.4 (48.0-62.9)	<.001	24.0 (17.6-30.4)	18.3 (12.5-24.1)	.03	36.6 (28.4-44.8)	25.7 (19.2-32.3)	.002

Abbreviations: FDA, US Food and Drug Administration; OB/GYN, obstetrics and gynecology.

^a^ A 29-year-old female cigarette smoker who smokes 10 cigarettes per day and has never tried any cessation medications or behavioral counseling for tobacco use.

^b^ A 65-year-old male cigarette smoker who smokes 40 cigarettes per day and has had multiple unsuccessful quit attempts with various FDA-approved cessation medications.

## Discussion

In this survey study, nearly 70% of physician respondents reported being asked about e-cigarettes by their patients, and 1 of 3 reported being asked in the past 30 days, which increased from 2018 to 2019 when controlling for physician specialty. In addition, 22% had recommended e-cigarettes at 1 time. These estimates could increase if the evidence that e-cigarettes are effective for cigarette smoking cessation strengthens.^10^

Consistent with prior research, primary care physicians and pulmonologists^14^ as well as cardiologists were more likely to be asked about e-cigarettes, whereas oncologists were least likely. Patients may feel reluctant to disclose smoking to oncologists due to stigma and guilt.^34^ There was low engagement with the PHS guideline, but physicians who implement the guidelines were more likely to be asked about e-cigarettes. Patients who smoke may feel more comfortable discussing e-cigarettes with physicians who have been previously supportive of cessation efforts. Physicians who were ever cigarette smokers were more likely to recommend e-cigarettes, perhaps because they may understand the difficulty in quitting.

Higher rates of recommending e-cigarettes were also noted for pulmonologists, consistent with prior literature,^14^ and cardiologists, but this finding was not significant when patient prompting was controlled for. Being asked about e-cigarettes by their patients was by far the most common factor associated with physicians recommending e-cigarettes. In a prior study, physicians were also more likely to engage in evidence-based tobacco treatment if the patient had prompted treatment from the physician.^35^

This study also examined hypothetical clinical scenarios with 2 different patients. Physicians were more likely to recommend e-cigarettes to an older heavy smoker with multiple failed quit attempts and more likely to advise FDA-approved medications to a younger light smoker who has never tried pharmacotherapy. When the risks of continued smoking outweighed the potential risk of e-cigarettes, physicians were more likely to offer e-cigarettes once other options were unsuccessful. In the absence of definitive evidence about e-cigarettes for cessation, it could be expected that physicians, who often find themselves in situations that may not be directly addressed in standard practice and treatment guidelines, took a pragmatic approach. This finding was consistent with previous qualitative studies showing that physicians were more inclined to recommend e-cigarettes to certain patients,^24,36^ including those who had multiple unsuccessful quit attempts.^24^

The current findings also highlighted misperceptions of the relative harm of e-cigarettes in comparison with other tobacco products, with 60% of physician respondents believing that all tobacco products were equally harmful. This belief is refuted by the growing body of evidence that combusted tobacco products (eg, cigarettes and cigars) contribute overwhelmingly to the societal harm of tobacco. While there is no evidence to date suggesting that e-cigarettes are completely harmless, numerous studies suggest that the level of toxins found in these products are orders of magnitude lower than those found in combusted tobacco.^2,22,37^ Other studies have demonstrated misperceptions by physicians regarding other important tobacco control issues, such the harm of nicotine.^38,39^ Based on the growing importance of nicotine as a central component of a potential FDA regulatory framework,^40^ it is critical to address physician nicotine misperceptions and to correct misperceptions regarding the relative harm of various tobacco products as more modified-risk tobacco products may be introduced through an FDA authorization process. These results suggest that physician education regarding harm reduction is warranted and should be a component of this future strategy. The results also suggested that physicians who already endorse a risk continuum were more likely to recommend e-cigarettes to their patients.

### Strengths and Limitations

This study had many strengths, including a large sample size, diverse physician characteristics, and established instruments. This study also had limitations. First, data are self-reported and may be subject to self-report bias. In addition, while we obtained fairly high response rates, nonresponse bias is a possibility, and topic salience is 1 of the strongest estimators of survey response. However, there were no significant differences between respondents and nonrespondents within specialties with respect to mean age and sex with the exception of oncologists; nonresponding oncologists were on average 2 years older than oncologists who responded to the survey. Second, because sampling probabilities varied by specialty, the overall sample was not nationally representative but was randomly drawn, and analyses were adjusted by specialty. Third, for our clinical scenarios, 2 sexes were presented to the physicians. It is possible that differences noted between the older heavier smoker with failed pharmacotherapy-assisted quit attempts and the younger light smoker with no experience with pharmacotherapy maybe confounded by sex.

## Conclusions

With many patients inquiring about e-cigarettes, it is important to understand the perceptions and recommendation practices of physicians as they are a trusted source of health information and are facilitators to tobacco use cessation. e-Cigarettes may play a pivotal role in the new FDA nicotine policy framework and thus may affect tobacco use patterns throughout the country. These findings suggest that some physicians believe e-cigarettes could help patients quit smoking in certain circumstances but may require more evidence regarding their safety and effectiveness. Additionally, more than half of the physicians believed that all tobacco products are equally harmful, and this belief was associated with lower rates of recommending e-cigarettes. As the evidence base grows for e-cigarette efficacy for smoking cessation, physicians’ understanding of e-cigarettes in the context of harm reduction must keep pace with the emerging scientific evidence through effective educational opportunities. Such opportunities should address e-cigarette safety and efficacy and correct misperceptions that all tobacco products are equally harmful.
